# Ectopic Calcification: What Do We Know and What Is the Way Forward?

**DOI:** 10.3390/jcm12113687

**Published:** 2023-05-26

**Authors:** Birgitta M. G. Snijders, Mike J. L. Peters, Huiberdina L. Koek

**Affiliations:** 1 Department of Geriatrics, University Medical Center Utrecht, Utrecht University, 3584 CX Utrecht, The Netherlands; 2 Department of Internal Medicine, University Medical Center Utrecht, Utrecht University, 3584 CX Utrecht, The Netherlands

Ectopic calcification, or ectopic mineralization, is a pathologic condition in which calcifications develop in soft tissues [1]. The spectrum of ectopic calcification disorders is broad, ranging from common vascular calcification associated with cardiovascular disease to rare hereditary calcification disorders affecting skin, eyes, brain parenchyma, kidneys, or cartilage [2,3]. The clinical presentation of ectopic calcification depends on the localization of the calcific deposits. Vascular calcification may result in ischemic heart disease, hypertension, cardiac hypertrophy, or peripheral arterial disease [4]. Vascular calcification is a highly prevalent problem associated with aging, chronic kidney disease, and diabetes mellitus [1,5,6,7,8]. In rare disorders, for example, in Pseudoxanthoma Elasticum (PXE), a systemic calcification disease, visual impairment or skin plaques can occur [9]. In Primary Familial Brain Calcification (PFBC), another rare calcification disease in the basal ganglia, patients develop symptoms consisting of cognitive impairment, movement disorders, and neuropsychiatric problems [10]. Although the disease burden of ectopic calcification is significant, a large knowledge gap exists, as the molecular pathogenesis is still poorly understood, and no causal treatment is available [11].

The pathophysiology of ectopic calcification appears to be similar to the physiological process of bone tissue mineralization [4]. Ectopic calcification was initially thought to be a passive and degenerative process. However, current evidence suggests it is a highly complex mechanism that results in mineralization [6]. Calcification is normally restricted to hard tissues such as bone, teeth, and cartilage [3]. In short, bone formation is a two-step process that involves the secretion of an extracellular matrix by osteoblasts and the subsequent mineralization of that matrix by crystalline hydroxyapatite formation, which is an accumulation of inorganic phosphate (Pi) and calcium ions [12]. In physiological circumstances, several regulatory processes prevent the formation of calcific deposits in soft tissues [3]. A disturbance in these regulatory processes may result in pathological ectopic calcification. These mechanisms are complex and partially overlapping, and their initial drivers are unclear [11]. Mechanisms contributing to soft tissue mineralization include the induction of bone formation, differentiation of vascular smooth muscle cells into an osteogenic phenotype, oxidative stress, apoptosis, mitochondrial dysfunction, mechanical stress, disbalance in calcium–phosphate homeostasis, and loss of inhibitors [4,6,8,11,12,13,14,15]. Ultimately, a disbalance between these calcification promotors and inhibitors results in the formation and deposition of hydroxyapatite crystals, which leads to calcific lesions [8]. Promotors of the calcification process include, for instance, calcium and Pi. A powerful inhibitor is inorganic pyrophosphate (PPi) [4,6,12,16]. 

Studies in hereditary ectopic calcification disorders have been instrumental in identifying the pathophysiological mechanisms of ectopic mineralization [17]. If a single genetic mutation can result in multisystem calcifications, identifying its exact function will help to unravel mechanisms through which the development of ectopic calcification occurs in other diseases. PXE is often seen as the prototype for hereditary ectopic mineralization diseases [18]. A loss-of-function mutation in the ABCC6 gene causes PXE. ABCC6 encodes for an adenosine triphosphate (ATP) binding efflux transporter, which facilitates the transport of ATP into the systemic circulation, where it is converted into adenosine monophosphate (AMP) and PPi by the enzyme ENPP1 [3,17,18]. A mutation in the ABCC6 gene is associated with low PPi levels [17,18]. PPi normally directly inhibits the accumulation of calcium and phosphate, preventing hydroxyapatite crystals from forming in soft tissues [15]. Low levels of PPi are found in PXE patients, which illustrates the critical role PPi has in preventing ectopic calcification [19]. Other PPi deficiency syndromes include General Arterial Calcification of Infancy (GACI) and arterial calcification due to CD73 deficiency (ACDC) [18]. GACI is a severe ectopic calcification disorder primarily affecting the cardiovascular system and is often diagnosed prenatally by routine ultrasound. The disease is caused by either a mutation in the ABCC6 gene or ENPP1 gene, the latter encoding for the enzyme that hydrolyzes ATP to AMP and PPi [18,19]. In ACDC, a genetic mutation in the NT5E gene reduces CD73 levels. CD73 normally converts AMP to adenosine and Pi. The proposed mechanism is that a reduction in extracellular adenosine levels enhances the enzyme tissue-nonspecific alkaline phosphatase (TNAP) activity, subsequently degrading PPi to Pi [15,20]. Thus far, in patients with PFBC, six genes have been identified to cause brain calcification. These genes result in an imbalance of Pi, a disruption of the blood–brain barrier integrity and dysfunctional pericyte maintenance, or both [21]. A mutation in the SLC20A2 gene, which encodes for the type III sodium-dependent Pi transporter 2 (PiT2), causes Pi to accumulate in the vascular extracellular matrix. A mutation in the XPR1 gene, which encodes for a Pi exporter, results in higher concentrations of intracellular Pi [21]. Remarkably, although the functions of these genes appear to be contradictory, both genetic mutations result in brain calcification. Intracellular Pi plays an essential role in ATP synthesis, while elevated levels of serum Pi are associated with hydroxyapatite formation [15,21]. The PDGFRB gene, which plays an important role in maintaining the blood–brain barrier, also regulates the phosphate transporter PiT1 [21]. Unfortunately, our understanding of the development of brain calcifications is far from complete. A schematic overview of the proposed mechanisms of ectopic calcification in rare hereditary calcification disorders is shown in Figure 1. These rare disorders illustrate the complexity of the pathophysiological processes of ectopic calcification and emphasize the crucial role of Pi and PPi homeostasis.

Another challenge is posed by how to detect and quantify ectopic calcification. A range of noninvasive and invasive techniques have been applied to assess calcific deposits [22]. CT is nowadays considered the golden standard for identifying vascular mineralization in vivo [22]. Several methods have been developed to compute the calcification load, of which the Coronary Artery Calcium score is the most well known. This score predicts the risk of coronary artery disease in both asymptomatic and symptomatic individuals [23]. In order to quantify brain calcifications, Nicolas et al. developed the Total Calcification Score [24]. More research is needed to validate these scores further, and other methods to quantify ectopic calcification, such as using volume or mass measurements, should be invested in as well. Other diagnostic techniques include MRI, plain radiography, ultrasound, PET scan, ankle–brachial index, angiography, and histology [22]. Most imaging techniques fail to detect microcalcifications and often cannot distinguish intimal from medial vascular calcification [11,22]. Although there is some overlap in the pathophysiology of intimal and medial calcification, their clinical implications are different. Intimal calcification is strongly associated with atherosclerosis and arterial obstruction, such as myocardial infarction, while medial calcification is linked to vessel stiffness [13,25]. Furthermore, there is a growing interest in serum biomarkers that assess the presence and extensiveness of ectopic calcification. For example, an in vitro blood test has recently been developed to measure the calcification propensity in serum (T50 test). Several studies have demonstrated an independent association between the T50 test and cardiovascular mortality [26,27]. One study showed that T50 is associated with PXE disease severity [28]. However, no significant association was found between the serum T50 test and cardiovascular events in the general population or total body CT calcium score in PXE patients [26,28]. Further research is warranted before implementation of this test in clinical practice. Numerous other biomarkers have been investigated, but no ideal diagnostic marker has been identified yet [29]. Adequate detection and quantification of ectopic calcification and its biomarkers remain a challenge.

Currently, no causal treatment options exist which effectively halt or diminish ectopic calcification [6]. However, several pharmacological agents have been investigated that show promising results in slowing disease progression [6]. Phosphate binders and calcimimetic agents such as cinacalcet, sodium thiosulfate, and vitamin K may decelerate vascular calcification [6,11]. Most trials were performed in specific patient groups, such as in patients with chronic kidney disease or on hemodialysis [11]. Nonetheless, a recent systematic review evaluating therapeutic interventions for vascular calcification in patients with chronic kidney disease concluded that the evidence is either insufficient or conflicting [30]. A novel approach in the field of hereditary ectopic calcification disorders is treatment with bisphosphonates. Bisphosphonates are analogs of PPi, a strong inhibitor of mineralization (Figure 1) [31]. A systematic review showed that bisphosphonates reduce arterial wall calcification [31]. Over the last few years, several studies have evaluated the effects of bisphosphonate therapy in genetic diseases such as PXE and GACI [9,32,33,34]. For example, a randomized-controlled trial performed in PXE patients concluded that treatment with etidronate, a first-generation bisphosphonate, halts systemic arterial calcification [34]. Currently, several trials are being undertaken to assess the effects of etidronate in patients with ACDC (NCT01585402), PXE [35], and PFBC (NCT05662111). A particular interest is taken in etidronate due to its close chemical structure to PPi [36]. However, a treatment option that effectively resolves ectopic calcification remains yet to be established. Due to the highly complex pathophysiology of ectopic calcification, individually targeted therapies might be needed.

In conclusion, ectopic calcification is a common problem with a high disease burden. A deeper understanding of the complex and broad spectrum of ectopic calcification syndromes is warranted. Much regarding the pathophysiology is still unknown, and many challenges arise in diagnosing and treating this heterogenous clinical syndrome. Research in rare hereditary ectopic calcification disorders can help achieve a more complete understanding.

## Figures and Tables

**Figure 1:**
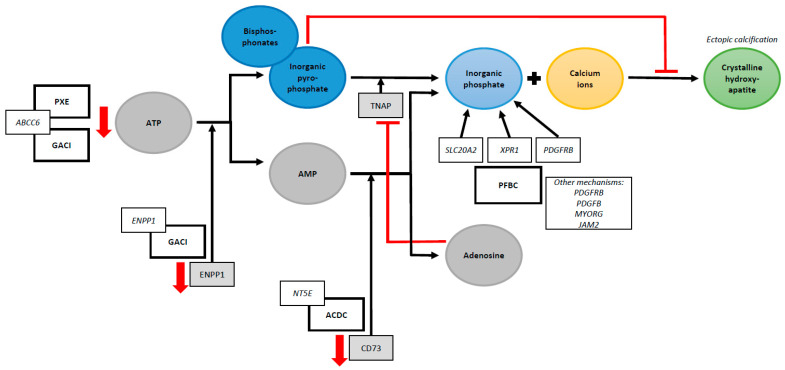
Schematic overview of proposed mechanisms of ectopic calcification in rare hereditary calcification disorders. Abbreviations: PXE = Pseudoxanthoma Elasticum, ABCC6 = ATP Binding Cassette Subfamily C Member 6, GACI = General Arterial Calcification of Infancy, ATP = Adenosine Triphosphate, ENPP1 = Ectonucleotide Pyrophosphatase/Phosphodiesterase 1, AMP = Adenosine Monophosphate, NT5E = 5’-Nucleotidase Ecto, ACDC = Arterial Calcification due to CD73 Deficiency, TNAP = Tissue-Nonspecific Alkaline Phosphatase, PFBC = Primary Familial Brain Calcification, SLC20A2 = Solute Carrier Family 20 Member 2, XPR1 = Xenotropic And Polytropic Retrovirus Receptor 1, MYORG = Myogenesis Regulating Glycosidase (Putative), JAM2 = Junctional Adhesion Molecule 2, PDGFB = Platelet-Derived Growth Factor Subunit B, and PDGFRB = Platelet-Derived Growth Factor Receptor Beta.
